# HLF and hTERT cooperatively enable partial immortalization of human hematopoietic stem and progenitor cells

**DOI:** 10.3389/fbioe.2025.1731355

**Published:** 2026-01-12

**Authors:** Manoj Kumar K. Azhagiri, Els Verhoeyen, Kumarasamypet Murugesan Mohankumar, Srujan Kumar Marepally, Vigneshwaran Venkatesan, Saravanabhavan Thangavel

**Affiliations:** 1 Centre for Stem Cell Research (CSCR), A Unit of BRIC-InStem Bengaluru, Christian Medical College Campus, Vellore, Tamil Nadu, India; 2 Manipal Academy of Higher Education, Manipal, Karnataka, India; 3 CIRI–International Center for Infectiology Research, Inserm, U1111, Université Claude Bernard Lyon 1, CNRS, UMR5308, Ecole Normale Supérieure de Lyon, Université Lyon, Lyon, France; 4 Université Côte d'Azur, INSERM, Nice, France

**Keywords:** Ex vivoHSPC culture, hematopoietic stem and progenitor cells, HLF, immortalization, primary cell-like models, prolonged HSPCs culture, HSPCs immortalization

## Abstract

Hematopoietic stem and progenitor cells (HSPCs), residing at the apex of the hematopoietic hierarchy, are critical for the generation of all blood and immune cell lineages. This unique capacity makes HSPCs indispensable for advancing cell and gene therapies aimed at correcting defects across hematopoietic lineages. However, current gene therapy development is constrained by the requirement for fresh primary HSPCs, hindering the breadth of preclinical validation. While several hematopoietic lineages have been immortalized to facilitate research, the stable immortalization of human HSPCs remains unreported. Here, we demonstrate that combinatorial overexpression of HLF, a key regulator of stem cell maintenance, and hTERT, a telomere maintenance factor in primitive human HSCs, supported by BaEV-mediated transduction and optimized culture conditions, yields partial immortalization of HSPCs. These genetically modified cells sustained for up to 70 days and exhibited limited differentiation towards erythroid, megakaryocytic, and macrophage lineages. Our model establishes a protocol for generating primary cell like context for testing gene therapy strategies, enabling functional assessment in both undifferentiated HSPCs and their lineage-committed progeny.

## 1 Introduction

The mobilization of hematopoietic stem and progenitor cells (HSPCs) from their native bone marrow microenvironment into the peripheral bloodstream, followed by their isolation from other blood components, forms the cornerstone of contemporary hematopoietic stem cell transplantation (HSCT). This well-established protocol not only underpins HSCT as one of the most widely practiced cell therapies but also facilitates significant advancements in gene therapy and regenerative medicine (Naldini, 2019). Beyond the tremendous progress in clinical protocols, there remains a critical demand for a continuous and reliable source of differentiated blood cells such as red blood cells (RBCs) and platelets for transfusion purposes. *Ex vivo* differentiation of HSPCs into mature blood products could address transfusion needs and expedite the preclinical evaluation of novel gene therapy constructs targeting hematological disorders. Current approaches to generating hematopoietic lineage cells from induced pluripotent stem cells (iPSCs) through reprogramming and subsequent differentiation to HSPCs are often burdened by lengthy procedures and high production costs, limiting their practical use (Ng et al., 2024). Meanwhile, the *ex vivo* expansion of primary HSPCs using small molecule cocktails is being explored in clinical trials, but these approaches have yet to achieve sustained long-term expansion or effectively prevent differentiation during *ex vivo* culture (Milano et al., 2023; Sakurai, 2023). Additionally, sourcing HSPCs for research purposes presents considerable challenges, particularly in patients suffering from hematological diseases characterized by bone marrow inflammation or failure, such as sickle cell disease (SCD), β-thalassemia, and Fanconi anemia.

Immortalized HSPC lines could furnish an enduring and scalable supply of progenitor cells, greatly facilitating preclinical studies, drug screening, and gene therapy validation. Moreover, these stable cell lines might serve as a renewable source of differentiated blood products for transfusion medicine. On this basis, we set out to determine whether genetic modification could enable the immortalization of HSPCs, thereby providing a stable and renewable platform for research and therapeutic applications. Several studies have shown that the lentiviral mediated stable expression of human telomerase reverse transcriptase (hTERT) preserves normal cell physiology and has been successfully tested in various models including fibroblasts, MSCs and osteoblasts. Since HSPCs rapidly lose stemness *in vitro*, hTERT expression alone cannot ensure their prolonged maintenance. Strategies that combine telomere stabilization with factors promoting stemness and blocking differentiation are therefore essential. Recent findings identify hepatic leukemia factor (HLF) and myeloid/lymphoid or mixed-lineage leukemia (trithorax homolog, *Drosophila*); translocated to, 3 (MLLT3) as pivotal regulators of primitive HSPC populations, underscoring their relevance for immortalization approaches (Calvanese et al., 2019; Calvanese et al., 2022; Lehnertz et al., 2021). Hepatic Leukemia Factor (HLF), a member of the PAR bZIP transcription factor family, is highly expressed in long-term repopulating hematopoietic stem cells (HSCs) and plays a pivotal role in regulating their quiescence and self-renewal. By preventing premature exhaustion, HLF maintains the stem cell pool, with its overexpression in mouse and human models enhancing long-term engraftment, preserving stemness, and promoting sustained proliferation without immediate differentiation (Komorowska et al., 2017; Lehnertz et al., 2021). In contrast, MLLT3 (AF9), a chromatin regulator, interacts with the super elongation complex (SEC) and DOT1L to ensure transcriptional fidelity in HSPCs, stabilizing progenitor states and regulating myeloid/erythroid lineage priming, though it is less potent in driving self-renewal compared to HLF (Calvanese et al., 2019).

Building on this rationale, we genetically modified primitive CD90^+^ HSCs by overexpressing hTERT together with Doxycycline (Dox) induced conditional expression of HLF or MLLT3, which extended the culture period of HSPCs to 70 days.

## 2 Materials and methods

### 2.1 Isolation and purification of CD34^+^ HSPCs

The Residual granulocyte colony-stimulating factor (G-CSF) mobilized peripheral blood (mPB) product, remaining after allogeneic stem cell transplantation, was obtained from the Department of Hematology, Christian Medical College, Vellore, following Institutional Review Board (IRB) approval. The Peripheral blood mononuclear cells (PBMCs) were separated by lymphoprep density gradient centrifugation. The CD34^+^ cells from PBMNCs were isolated using the CD34^+^ selection kit (STEMCELL Technologies) and subsequently expanded using AC media or Hibernation medium. AC contains StemSpan™ SFEM II medium supplemented Stem cell factor (SCF, 240 ng/mL), FMS-like tyrosine kinase three ligand (Flt3-L, 240 ng/mL), Thrombopoietin (TPO, 80 ng/mL), and Interleukin-6 (IL-6, 40 ng/mL). For the hibernation medium, the cells are cultured in (SCF, 300 ng/mL), (IL11, 20 ng/mL), (L-Glutamine, 2 mM), and (2-Mercaptoethanol, 100 μM) (Oedekoven et al., 2021). Both the culture media were supplemented with small molecule cocktail of Resveratrol, Stem Reginin-1 and UM729 (RUS) as described in our previous work (Christopher et al., 2022). In both the conditions the cells were cultured at a confluency of 2 × 10^5^/mL. HSPCs were analyzed for cell-surface marker expression both immediately after purification and subsequent days of culture using a BD FACSAria™ III flow cytometer.

### 2.2 Flow based sorting of CD90^+^ HSCs

For sorting CD90^+^ cells from CD34^+^ HSPCs, the purified CD34^+^ cells were cultured with the above-mentioned cytokines for 16–24 h. Then the cultured cells were stained with the CD90^+^ antibody for 20 min at room temperature (8µg/1 × 10^6 cells). Following incubation, the cells were washed with PBS and CD90^+^ cells were sorted using the flow cytometer BD FACS ARIA III in purity mode.

### 2.3 Lentiviral production

On reaching 60%–80% confluency, transfect HEK-293T cells with 2 µg of the transgene plasmid (hTERT + HLF or hTERT + MLLT3), 1 µg of the packaging plasmid (psPAX2), and 1 µg of the envelope plasmid (VSV-G) using FuGENE at a 1:3 ratio in OptiMEM medium in 6 cm dish. The transfection mix were incubated for 15 min to allow complex formation. Add the mix dropwise to the cells immediately. For virus collection, harvest the media containing the virus at 48 and 72 h post-transfection, pool the collected media, and perform ultracentrifugation at 20,000rpm for 2 h to pellet the virus and resuspend the viral pellet in plain SFEM-II. Aliquot the virus and store at −80 °C. The plasmid PSJL224 was a kind gift from st jude childrens' research hospital.

### 2.4 Lentiviral transduction of CD90^+^ HSCs

Retronectin was coated onto 96-well plates according to the manufacturer’s instructions to facilitate efficient transduction. The transduction mix was prepared in SFEM-II medium by supplementing 6 mM of HEPES buffer and 10 μg/mL of Polybrene. CD90^+^ sorted cells were then resuspended in this transduction mix and the viral suspension was added to the wells. The plates were centrifuged at 800 *g* for 30 min at 24 °C to enhance viral contact with the cells, followed by incubation in a 5% CO_2_ incubator. After 24 h, viral washouts were performed to replace with fresh medium. The cells were cultured and analyzed based on experimental requirements. For testing the transduction enhancers, the following reagents were tested: Retronectin (50 μg/mL), Polybrene (10 μg/mL), Cyclosporin H (8 µM), LentiBoost (0.7 mg/mL), Protamine sulfate (5 μg/mL), and Prostaglandin E2 (PGE2; 10 µM).

### 2.5 Colony forming unit (CFU) assay

Based on experimental requirements, 500 HSPCs were seeded in the 1.5 mL of Methocult Optimum (STEMCELL Technologies). After 14 days of culture, hematopoietic progenitor colonies were scored based on morphology and categorized as Burst forming unit-erythroid (BFU-E), Colony forming unit-erythroid (CFU-E), Colony forming unit-granulocyte-monocyte progenitor (CFU-GM) and Colony forming unit-granulocyte, erythrocyte, monocyte, megakaryocyte (CFU-GEMM).

### 2.6 Erythroid differentiation

The erythroid differentiation protocol for HSPCs was adapted from our previously published protocol (Venkatesan et al., 2023). The three-stage protocol begins with phase I, where CD34^+^ cells are cultured at a density of 5 × 10^4 cells/mL from day 0 to day 8, with a media change on day 4. The phase I medium consists of IMDM GlutaMAX Supplement with 5% AB serum, insulin (20 μg/mL), heparin (2 U/mL), EPO (3 U/mL), holotransferrin (330 μg/mL), SCF (100 ng/mL), IL3 (50 ng/mL), and hydrocortisone (1 μg/mL). In phase II, from day 8 to day 12, cells are seeded at 2 × 10^5 cells/mL in medium identical to phase I but without hydrocortisone and IL3. In phase III, from day 12 to day 20, cells are cultured at 5 × 10^5 cells/mL in medium similar to phase II but excluding SCF, with a media change on day 16. On day 20, cells are harvested for quantifying the percentage of reticulocytes marked by CD235a^+^, Hoechst^−^.

### 2.7 Macrophage differentiation

Macrophage differentiation of HSPCs was performed following our previously published protocol (Karuppusamy et al., 2022). In brief, HSPCs were seeded in non-tissue culture-treated polystyrene plates using macrophage differentiation medium (SFEM-II supplemented with 100 ng/mL SCF, 50 ng/mL Flt3-L, 10 ng/mL IL-6, 10 ng/mL IL-3, 10 ng/ml GM-CSF, and 10 ng/ml M-CSF). Every 72 h, non-adherent cells were collected and replated in fresh macrophage differentiation medium. Adherent cells were maintained in RPMI medium with 10% FBS, 10 ng/ml GM-CSF, and 10 ng/ml M-CSF. After 14–16 days, adherent cells were examined under a microscope for morphological characteristics, detached using accutase and stained with antibody CD14. The stained cells were analyzed using a BD FACS Aria III flow cytometer.

### 2.8 Megakaryocyte differentiation

For megakaryocyte differentiation of immortalized HSCs, HSCs were seeded in megakaryocyte differentiation medium (SFEM-II supplemented with 100 uL of StemSpan™ Megakaryocyte Expansion Supplement (100X). Once in 3–4 days, cell were replenished with fresh media. After 14 days, cells were examined under a microscope for morphological characteristics, stained with antibody CD61 and CD41 for surface marker expression.

### 2.9 Quantitative real-time PCR analysis

48 h post transduction of hTERT, hTERT + HLF, and hTERT + MLLT3, a total of 1 × 10^6^ cells that were collected and RNA were extracted using RNeasy Mini Kit (Qiagen). 1μg of RNA were used for reverse transcription using Prime-script RT reagent kit (Takara Bio Inc.). Quantitative PCR was performed using SYBR Premix Ex Taq II (Takara Bio), and amplification was carried out on a QuantStudio 6 Flex Real-Time PCR System (Life Technologies). The primer sequences used for qPCR are listed in Supplementary Table S3.

## 3 Results

### 3.1 Design and evaluation of conditional immortalization systems regulated by doxycycline

**FIGURE 1:**
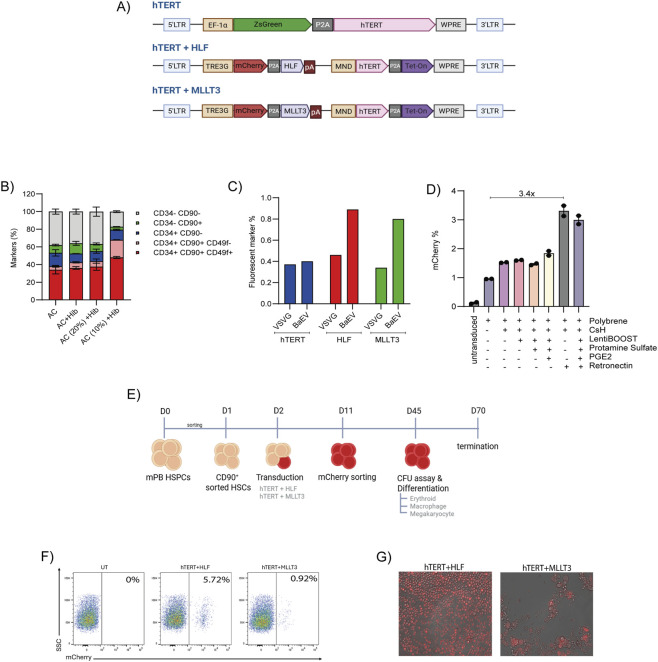
Transduction and selection of HSCs expressing the immortalization construct **(A)** Schematic representation of the plasmid constructs designed for constitutive hTERT expression alongside doxycycline-inducible expression of stemness related factors (n = 2). **(B)** Percentage of HSPC subsets analyzed by flow cytometry on day 21 of culture (n = 2, Donor = 1). The AC condition refers to cultures maintained in SFEM II medium supplemented with SCF, Flt3-L, TPO, and IL-6. The Hibernation (Hib) condition refers to cultures maintained in SFEM II medium containing SCF, IL-11, L-Glutamine, and 2-Mercaptoethanol. Both culture systems were maintained in the presence of Resveratrol, UM729, and SR1 **(C)** Transduction efficiency of GFP^+^ (hTERT) and mCherry^+^ (HLF and MLLT3) cells analyzed 48 h post-transduction in day 3 CD90^+^ sorted HSCs using different viral envelopes (n = 1, Donor = 1). **(D)** Evaluation of transduction enhancers in day 3 CD90^+^ sorted HSCs transduced with the MLLT3 construct packaged with the BaEV envelope, assessed 48 h post-transduction using flow cytometry (n = 2, Donor = 1). **(E)** schematic diagram of the experiment for immortalization in CD90^+^ sorted HSCs. **(F)** Representative flow cytometry plot showing the percentage of mCherry^+^ cells on day 10 post-transduction (n = 1, donor = 1). **(G)** Microscopy image of sorted day 19 mCherry ^+^ cells.

To generate immortalized HSPCs, we engineered three distinct constructs: hTERT, hTERT + HLF, and hTERT + MLLT3 (Figure 1A). In the hTERT construct, ZsGreen and hTERT were co-expressed under the constitutive EF1A promoter, separated by a P2A linker. For the hTERT + HLF and hTERT + MLLT3 constructs, we employed a bi-cistronic doxycycline-inducible system. In this design, hTERT and the Tet-On transactivator are driven by the constitutive MND promoter, while the TRE3G promoter controls the Dox inducible expression of mCherry together with HLF or MLLT3 separated by P2A linker. Upon doxycycline administration, TRE3G activation enables co-expression of mCherry and HLF (or MLLT3), allowing temporal control of stemness factor expression during the expansion phase versus the differentiation phase.

To validate construct designing, the hTERT was transduced into HEK-293T cells, and the proportion of GFP positive (GFP^+^) cells was quantified by flow cytometry 48 h post-transduction. Compared to untransduced controls, approximately 45.7% of cells were GFP^+^(Supplementary Figure S1A). Additionally, relative hTERT mRNA expression was significantly elevated compared to untransduced cells (Supplementary Figure S1B). Similarly, hTERT + HLF and hTERT + MLLT3 were transduced and the mCherry expression (35.2% and 32.1%) (Supplementary Figure S1C) was measured respectively 48 h following post transduction. Furthermore, relative mRNA expression levels of hTERT, HLF, and MLLT3, normalized to β-actin, were significantly increased in both constructs compared with untransduced cells (Supplementary Figure S1D,E). Since the latter two cassettes exploit the Dox inducible system, we wanted to check the impact of Dox on the stemness and the proliferation of HSPCs. The impact was evaluated by culturing HSPCs with doxycycline concentrations ranging from 0 to 1 μg/mL to assess potential toxicity on HSPC subsets marked by CD34^+^CD90^+^ cells. Flow cytometry analysis on day 5 of culture revealed no notable differences in the HSPCs subsets across doxycycline doses (0.25–1 μg/mL) compared to the control (0 μg/mL) (Supplementary Figure S1F). These findings demonstrate that doxycycline-regulated HLF and MLLT3 expression systems are suitable for conditional immortalization of HSPCs.

### 3.2 Optimization of culture and transduction in CD90^+^ HSCs

For immortalization, it is essential to target the HSPC fraction with the stemness and long-term repopulating potential, rather than the bulk population, to ensure that immortalized cells preserve stem cell properties. Among HSPC subsets, CD34^+^CD90^+^ cells are well recognized for their robust and durable engraftment capacity. Equally critical is the establishment of culture conditions that prevent exhaustion and sustain stemness over extended periods. To address this, we initiated our studies by sorting CD90^+^ HSCs 16–24 h after CD34^+^ cell isolation from peripheral blood mononuclear cells (PBMNCs). These cells were then cultured in hibernation medium (hib), which has recently been reported to support the maintenance of long-term Cord blood (CB) derived HSCs *in vitro* (Oedekoven et al., 2021). We subsequently compared three conditions including our previously established AC + RUS medium [stem cell factor (SCF, 240 ng/mL), FMS-like tyrosine kinase three ligand (Flt3-L, 240 ng/mL), thrombopoietin (TPO, 80 ng/mL), and interleukin-6 (IL-6, 40 ng/mL) and small molecule cocktail of Resveratrol, Stem Reginin-1 and UM729 (RUS)] (Christopher et al., 2022), hibernation medium alone (Hib) [SCF 300 ng/mL, IL11 20 ng/mL, L-Glutamine 2mM, 2-Mercaptoethanol 100 μM], and hibernation medium supplemented with the RUS cocktail (Hib + RUS). Over an 8-day culture period, we observed distinct proliferation dynamics, while AC + RUS promoted robust expansion, whereas both Hib and Hib + RUS led to poor survival and diminished proliferative capacity (Supplementary Figure S1G) with similar frequency of CD34^+^ CD90^+^ cells (Supplementary Figure S1H). Since the hibernation protocol has been shown to preserve stemness in CB derived HSPCs, and given compelling evidence from the literature (Sakurai et al., 2023) that FLT3, and TPO signaling pathways are critical for the survival of mobilized peripheral blood (mPB) HSPCs, we supplemented the hibernation medium with cytokines at 100%, 20%, and 10% of the standard AC concentration. We then cultured CD90^+^ cells for 21 days. By day 21, analysis of HSPC subsets revealed that the optimized culture particularly the 10% cytokine condition in combination with hibernation medium retained the highest proportion of CD34^+^CD90^+^ CD49f^+^ and CD34^+^CD90^+^ CD49f^−^ cells (Figure 1B; Supplementary Figure S2A). This suggests that the medium helps mitigate exhaustion by limiting proliferation (Supplementary Figure S2B). Importantly, the colony-forming potential of cells expanded under these conditions for 21 days remains unaffected compared to standard conditions (Supplementary Figure S2C).

We next evaluated viral envelopes for their ability to efficiently transduce CD90^+^ HSCs. Given prior evidence that the Baboon envelope (BaEV) enhances transduction of primitive HSCs (Bernadin et al., 2019), we compared its performance against the conventional VSV-G envelope. As expected, VSV-G achieved higher transduction efficiency in HEK293T cells (Supplementary Figure S2D). However, in CD90^+^ HSCs, the BaEV envelope outperformed VSV-G (Figure 1C). Despite this improvement, overall transduction efficiency in HSCs remained low, prompting us to test a panel of transduction enhancers (Figure 1D). The combination of retronectin coating, polybrene, and cyclosporine-H proved most effective, yielding a 3.4-fold increase in transduction efficiency relative to polybrene alone.

### 3.3 Partial immortalization of healthy donor HSPCs

**FIGURE 2:**
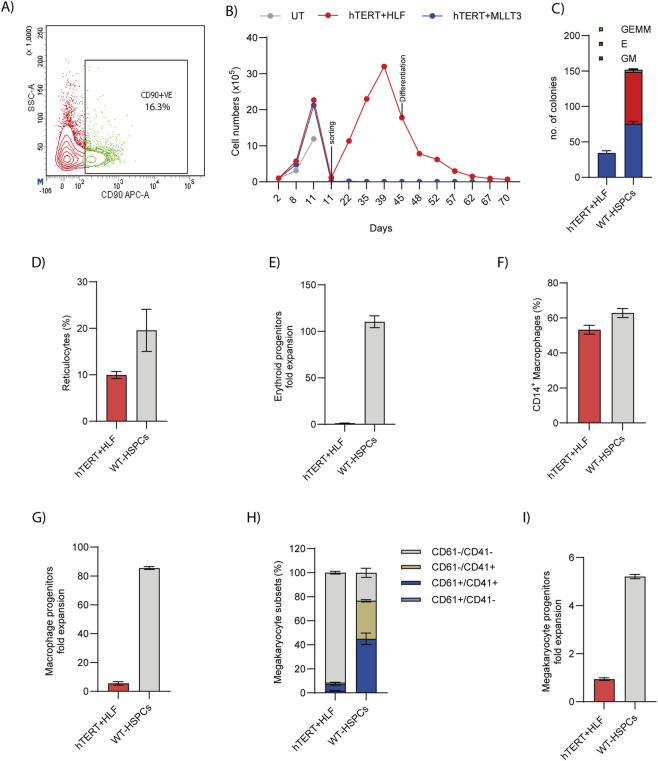
Partial immortalization of healthy donor HSPCs **(A)** Representative flow cytometry dot plot showing the sorting gate for the CD90^+^ population. **(B)** Expansion kinetics of mCherry^+^ cells sorted on day 11 and subsequently cultured for up to 70 days (n = 1, donor = 1). **(C)** Number of CFUs generated from day 40 HSPCs and wild type (WT) HSPCs (n = 2, donor = 1). **(D)** Percentage of reticulocytes produced under erythroid differentiation conditions (n = 2, donor = 1). **(E)** Fold expansion of erythroblasts during erythroid differentiation *in vitro* (n = 2, donor = 1). **(F)** Percentage of CD14^+^ macrophages generated from day 40 expanded HSPCs on *in vitro* macrophage differentiation, compared to day 3 wildtype HSPCs (n = 2, donor = 1). **(G)** Fold expansion of macrophage progenitors following differentiation (n = 2, donor = 1). **(H)** Percentage of megakaryocytes derived from day 40 expanded HSPCs under *in vitro* megakaryocyte differentiation, compared to day 3 wildtype HSPCs (n = 2, donor = 1). **(I)** Fold expansion of megakaryocyte progenitors during *in vitro* differentiation (n = 2, donor = 1).

After optimizing culture and transduction conditions, we transduced CD90^+^sorted HSCs from a healthy donor with either hTERT + HLF or hTERT + MLLT3 for immortalizing HSPCs (Figure 1E). Flow cytometry analysis on day 10 post-transduction revealed 5.72% mCherry^+^ cells in the hTERT + HLF group, compared with only 0.92% in the hTERT + MLLT3 group (Figure 1F). Given the relatively low transduction efficiency, we enriched the mCherry^+^ fraction by sorting at day 11 to better assess the effects. Sorted cells expressing hTERT + HLF expanded rapidly and maintained proliferation for up to 39 days *in vitro*, followed by a gradual decline that extended to approximately 70 days (Figures 2A,B). In contrast, the mCherry^+^ fraction transduced with hTERT + MLLT3 failed to sustain long-term growth, instead showing progressive differentiation and cell death. As expected, control untransduced HSPCs exhibited limited expansion, highlighting the partial immortalization effect of the hTERT + HLF system. Microscopic inspection on day 19 further confirmed that hTERT + HLF transduced cells retained a small, round, stem cell-like morphology, consistent with their preserved stemness, whereas cells expressing hTERT + MLLT3 did not adopt this morphology and instead exhibited features of apoptosis or differentiation into committed progenitors (Figure 1G).

By day 40 post-expansion, the colony forming capacity of hTERT + HLF transduced cells were reduced compared with wild-type fresh HSPCs (Figure 2C), indicating a loss of functional progenitor activity associated with prolonged proliferation. To evaluate their differentiation competence, day 45 expanded cells were differentiated into erythroid, macrophage, and megakaryocyte lineages. These cells successfully generated terminally differentiated reticulocytes at detectable levels (Figure 2D), confirming retained terminal differentiation potential. However, assessment of erythroid progenitor fold expansion revealed that the hTERT + HLF system failed to generate larger numbers of cells, despite supporting progression to terminal differentiation (Figure 2E). Differentiation toward the myeloid lineage generated comparable percentages of CD14^+^ macrophages (Figure 2F), indicating that myeloid output capacity was largely preserved during extended culture. However, the fold expansion of macrophage progenitors was markedly reduced in the hTERT + HLF group (Figure 2G). Similarly, megakaryocyte differentiation of the hTERT + HLF cells yielded lesser proportion of terminally differentiated cells marked by CD61^+^/CD41^+^ (Figure 2H) and lower progenitor expansion (Figure 2I) compared to the wild-type HSPCs. Together, these results demonstrate that expression of hTERT combined with inducible HLF supports the partial immortalization of healthy donor HSPCs with limited differentiaion potential. Expanded cells maintained proliferative capacity for over 2 months in culture while partially retaining multilineage differentiation potential across erythroid, myeloid, and megakaryocytic pathways. The partial preservation of both progenitor expansion and functional output highlights this strategy as a promising approach, however further optimization are required for generating long-lived, functional HSPCs *in vitro.*

## 4 Discussion

In this study, we demonstrate that enforced expression of hTERT in combination with doxycycline-inducible HLF enables partial immortalization of primary human HSPCs derived from healthy donors. While hTERT alone extends proliferative capacity by preventing telomere shortening, it is insufficient to maintain stemness, consistent with prior studies in fibroblasts and mesenchymal stem cells where telomerase activity delays senescence but does not preserve lineage identity (Weng et al., 2022). By pairing hTERT with HLF, a transcription factor enriched in long-term repopulating HSCs, we cultured the HSPCs for over 70 days while retaining multilineage differentiation potential across erythroid, myeloid, and megakaryocytic lineages. These findings highlight the importance of combining telomere maintenance with stemness-enforcing transcriptional regulators to extend the utility of primary HSPCs *ex vivo*. Our results also reveal a clear functional divergence between HLF and MLLT3 in regulating HSPC behavior. Although both have been implicated in the preservation of primitive HSPC states, HLF consistently drove stronger proliferation compared with MLLT3 when co-expressed with hTERT. This is in line with reports identifying HLF as a master regulator of HSC quiescence and self-renewal, directly activating transcriptional programs that preserve stem cell identity and survival. In contrast, MLLT3 acts predominantly as a transcriptional stabilizer through interactions with the super elongation complex, thereby maintaining progenitor fidelity but not actively promoting self-renewal. The reduced expansion observed with hTERT + MLLT3 thus reflects its limited capacity to enforce stemness compared to HLF.

Importantly, the partially immortalized HSPCs generated here in part retained their functional capacity to differentiate into erythroid cells, macrophages, and megakaryocytes. The ability to produce reticulocytes and expand erythroblasts, generate CD14^+^ macrophages, and differentiate into CD61^+^/CD41^+^ megakaryocytes indicates that these cells remain competent to generate clinically relevant blood cell types after prolonged culture. This functional retention is critical for their potential use as a surrogate primary cell–like model for gene therapy validation and drug screening. The optimization of culture conditions and vector pseudotyping further enhances the robustness of this system. Our findings show that BaEV-pseudotyped lentiviral vectors mediated increased transduction efficiency in CD90^+^ HSCs compared with VSV-G support the growing recognition that receptor availability dictates vector tropism in primary stem cells. Additionally, the use of transduction enhancers such as retronectin, polybrene, and cyclosporine-H (Valeri et al., 2024) substantially increased efficiency, providing practical strategies for achieving reliable gene delivery into otherwise challenging primary HSPCs. Despite these encouraging findings, this study has several limitations. First, the experiments were conducted using a limited donor pool, which may restrict the generalizability of the results. Second, although proliferation and multilineage differentiation were observed *in vitro*, both the fold expansion and the degree of differentiation were lower than those achieved by wild-type HSPCs, accompanied by a pronounced myeloid bias in CFU assays. Finally, *in vivo* engraftment studies will be required to determine whether partially immortalized HSPCs retain durable long-term reconstitution capacity. Although, while HLF + hTERT supports partial immortalization, cells eventually showed signs of differentiation or apoptosis, indicating that additional factors or fine-tuned culture systems may be required to achieve stable, indefinite expansion.

This model provides a foundation for developing renewable, primary cell–like HSPC platforms that can accelerate preclinical testing of gene therapy strategies for hematological disorders. Future work could explore the integration of other stemness-associated transcription factors (e.g., HOXB4, MEIS1) with hTERT, or the application of small molecule modulators that mimic niche-like cues to further enhance stability. Moreover, adaptation of this system for patient-derived HSPCs could support disease modeling and personalized therapeutic testing. In conclusion, our findings establish that hTERT and HLF cooperatively extend the proliferative lifespan of human HSPCs while preserving multilineage differentiation potential, representing a critical step toward the generation of renewable HSPC resources. This approach addresses a longstanding barrier in hematopoietic research and holds promise for advancing gene therapy development and regenerative hematology.

## 5 Conclusion

This study demonstrates that co-expression of hTERT and HLF in human CD90^+^ HSCs enables partial immortalization. hTERT maintains telomere integrity, while HLF enhances stemness-associated transcriptional programs, together extending the proliferative lifespan of HSPCs in culture for up to 70 days. These modified cells retained the ability to differentiate into erythroid, myeloid, and megakaryocytic lineages, indicating that prolonged proliferation does not fully impair developmental potential. Optimization of BaEV-pseudotyped vectors and supportive culture conditions further improved gene delivery efficiency and preservation of primitive HSPC states. Although full immortalization and long-term engraftment remain goals for future work, this study lays a foundational framework for generating renewable, primary cell–like models of human hematopoiesis. These models hold immediate promise for preclinical gene therapy testing, disease modeling, and functional genomics research aimed at advancing regenerative hematology.

## Data Availability

The raw data supporting the conclusions of this article will be made available by the authors, without undue reservation.

## Supplementary material

SUPPLEMENTARY FIGURE S1 Refinement of culture and transduction strategies for HSPC immortalization **(A)** Percentage of GFP ^+^ cells quantified by flow cytometry 48 h following transduction with the hTERT construct (n = 3). **(B)** Relative mRNA expression levels of hTERT measured 48 h post-transduction, normalized to β-actin. **(C)** Percentage of mCherry ^+^ cells quantified by flow cytometry 72 h following transduction with the hTERT + HLF and hTERT + MLLT3 construct. **(D)** Relative mRNA expression levels of hTERT and HLF measured 48 h post-transduction of hTERT + HLF construct, normalized to β-actin (n = 3). **(E)** Relative mRNA expression levels of hTERT and MLLT3 measured 48 h post-transduction of hTERT + MLLT3 construct, normalized to β-actin (n = 3). **(F)** Percentage of HSPCs subsets analyzed by flow cytometry on Day 5 of culture with varying doses of Dox (n = 2, Donor = 1). **(G)** Proliferation kinetics of CD90⁺ sorted HSCs cultured over 8 days in various media compositions (n = 3, Donor = 1). **(H)** Frequency of CD34⁺ CD90⁺ HSCs assessed by flow cytometry on day 8 of expansion (n = 3, Donor = 1).

SUPPLEMENTARY FIGURE S2 Optimization of culture and transduction conditions for efficient gene delivery into CD90⁺ HSCs. **(A)** Absolute cell numbers of individual HSPC subsets after 21 days of expansion in the indicated culture conditions. **(B)** Expansion kinetics of HSPCs cultured in different media formulations over a 21-day period (n = 2, Donor = 1). **(C)** CFU assay quantifying the number of colonies generated by cells expanded for 21 days in various media formulations (n = 4, Donor = 1). **(D)** Transduction efficiency measured by the percentage of GFP⁺ (hTERT) and mCherry⁺ (HLF and MLLT3) cells 48 h post transduction in HEK293T cells using different viral envelopes (n = 2).
