# Supplementary material for: HLF and hTERT cooperatively enable partial immortalization of human hematopoietic stem and progenitor cells
Source: Front Bioeng Biotechnol. 2026 Jan 12;13:1731355. doi: 10.3389/fbioe.2025.1731355 (PMC12833506; doi:10.3389/fbioe.2025.1731355)
Supplement: Supplementary file 1 [file Table1.docx]

**Table 1. List of reagents**

| **SI.NO** | **Reagents** | **Catalogue No.** | **Brand** |
| --- | --- | --- | --- |
| 1. | Lymphoprep | 18061 | STEMCELL Technologies |
| 2. | CD34+ selection kit | 17856 | STEMCELL Technologies |
| 3. | StemSpan™ SFEM II | 09655 | STEMCELL Technologies |
| 4. | Stem cell factor | CYT-255 | Prospecbio |
| 5. | FMS-like tyrosine kinase 3 ligand | CYT-331 | Prospecbio |
| 6. | Thrombopoietin | CYT-11788 | Prospecbio |
| 7. | Interleukin-6 | CYT-213 | Prospecbio |
| 8. | Interleukin-11 | CYT-214 | Prospecbio |
| 9. | Interleukin-3 | CYT-210 | Prospecbio |
| 10. | L-Glutamine | 25030081 | Thermo Fischer Scientific |
| 11. | 2-Mercaptoethanol | M-6250 | Sigma-Aldrich |
| 12. | Resveratrol | 72864 | STEMCELL Technologies |
| 13. | Stem Reginin-1 | 72344 | STEMCELL Technologies |
| 14. | UM729 | 72332 | STEMCELL Technologies |
| 15. | Retronectin | T100A | TakaraBio |
| 16. | Polybrene | TR-1003 | Sigma-Aldrich |
| 17. | Cyclosporin H | SML-1575 | Sigma-Aldrich |
| 18. | LentiBoost | SB-P-LV-101 | Revvity |
| 19. | Protamine sulfate | HY-107911 | Medchem Express |
| 20. | Prostaglandin E2 | HY-101952 | Medchem Express |
| 21. | Methocult Optimum | H4034 | STEMCELL Technologies |
| 22. | AB serum | 092930949 | MP Biomedicals |
| 23. | Insulin | I9278 | Sigma-Aldrich |
| 24. | Heparin | 0210193125 | MP Biomedicals |
| 25. | Human Recombinant Erythropoietin | - | Zydus |
| 26. | Holotransferrin | PRO-315 | Prospecbio |
| 27. | Hydrocortisone | Sigma-Aldrich | H0-888 |
| 28. | Granulocyte-macrophage colony-stimulating factor | CYT-308 | Prospecbio |
| 29. | Macrophage colony-stimulating factor | CYT-221 | Prospecbio |
| 30. | FBS | A5256701 | Thermo Fischer Scientific |
| 31. | RPMI medium | 11876093 | Thermo Fischer Scientific |
| 32. | Accutase | 07922 | STEMCELL Technologies |
| 33. | Megakaryocyte Expansion Supplement (100X) | 02696 | STEMCELL Technologies |

**Table 2. List of antibodies**

| **SI.NO** | **Antibodies** | **Catalogue No.** | **Brand** |
| --- | --- | --- | --- |
| 1. | Mouse Anti-Human CD34 | 348057 | BD Biosciences |
| 2. | Mouse Anti-Human CD90 | 559869 | BD Biosciences |
| 3. | Mouse Anti-human CD235a | 563666 | BD Biosciences |
| 4. | Hoechst | 62249 | Thermo Fischer Scientific |
| 5. | Mouse Anti-human CD14 | 555399 | BD Biosciences |
| 6. | Mouse Anti-human CD61 | 564174 | BD Biosciences |
| 7. | Mouse Anti-human CD41 | 555467 | BD Biosciences |

**Table 3. List of qPCR primers**

| **Name** | **Sequence** |
| --- | --- |
| RT_MLLT3_5F | TCTGGGTATGCTGGTTTCATTT |
| RT_MLLT3_3R | GGCCTTCAAGATGCAGGAATA |
| RT_HLF_5F | CCCTGAATCCCACCTTTATCC |
| RT_HLF_3R | CGGGCTGTTACTCTCATCATC |
| RT_hTERT_5F | GCCGATTGTGAACATGGACTACG |
| RT_hTERT_3R | GCTCGTAGTTGAGCACGCTGAA |
