# Supplementary figures and images for: HLF and hTERT cooperatively enable partial immortalization of human hematopoietic stem and progenitor cells
Source: Front Bioeng Biotechnol. 2026 Jan 12;13:1731355. doi: 10.3389/fbioe.2025.1731355 (PMC12833506; doi:10.3389/fbioe.2025.1731355)

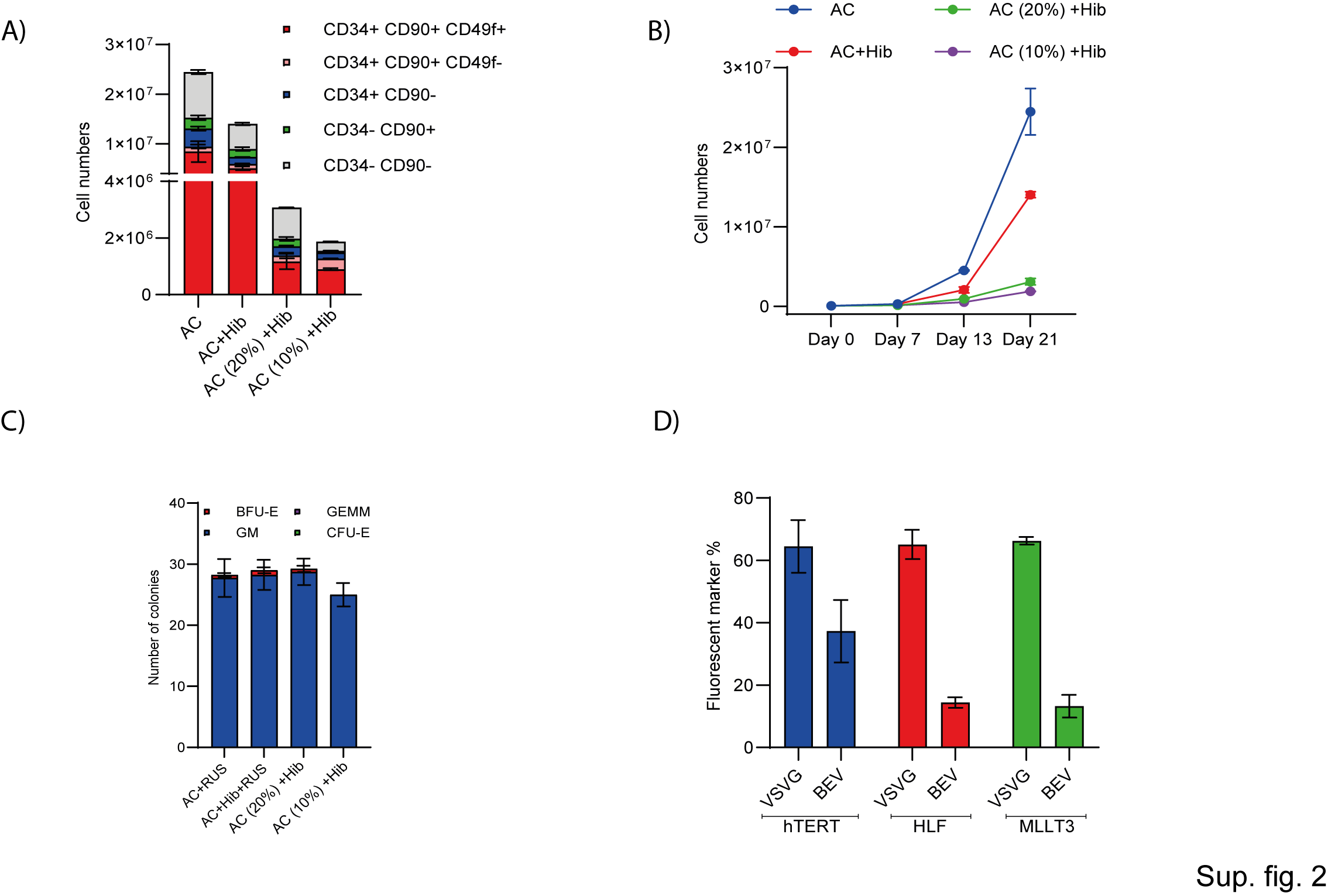

Supplement: Supplementary file 2 [file Image2.tif]

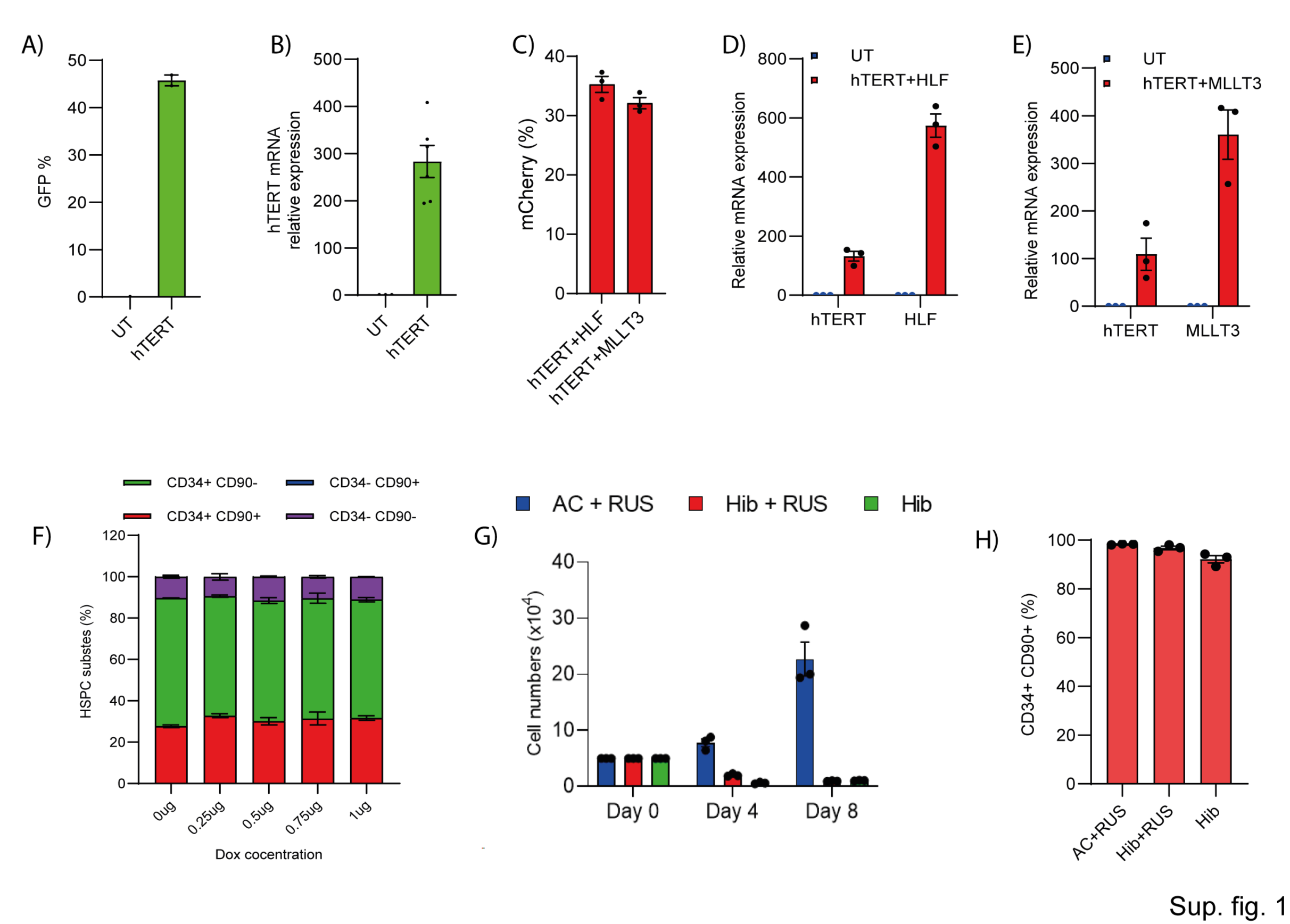

Supplement: Supplementary file 3 [file Image1.tif]
